# Hypophosphatasia: A Case of Two Patients With Spinal Cord Compression From Increase in Ligamentous Ossifications During Treatment

**DOI:** 10.1002/jbm4.10449

**Published:** 2021-03-05

**Authors:** Michel Laroche, Guillaume Couture, Marie Faruch, Adeline Ruyssen‐Witrand, Valérie Porquet‐Bordes, Jean Pierre Salles, Yannick Degboe

**Affiliations:** ^1^ Centre de Rhumatologie du Centre Hospitalo‐Universitaire de Toulouse [Rheumatology Center of Toulouse University Hospital] Toulouse France; ^2^ Service de Radiologie du Centre Hospitalo‐Universitaire de Toulouse [Department of Radiology of Toulouse University Hospital] Toulouse France; ^3^ Endocrinologie, Maladies Osseuses, Hôpital des Enfants, Centre de Référence des Maladies Rares du Métabolisme du Calcium et Phosphate, European Reference Network on rare bone diseases, Centre Hospitalo‐Universitaire de Toulouse Toulouse France; ^4^ INSERM UMR 1043 CNRS5825, Centre de Physiopathologie de Toulouse Purpan, Centre de Physiopathology Toulouse Purpan, Université de Toulouse Toulouse France

**Keywords:** BONE DISEASES, OTHER, THERAPEUTICS, OTHER, DISEASES AND DISORDERS OF/RELATED TO BONE, DISORDERS OF CALCIUM/PHOSPHATE METABOLISM

## Abstract

Treatment with asfotase alfa has transformed the prognosis of hypophosphatasia in children and improves the bone and muscle signs in adults. The doses used in adults are the same as in children, whereas bone remodeling is different between them. We report on the cases of two patients treated with 1 mg/kg/day of asfotase alfa who developed spinal cord compression from spinal ossifications during treatment. The first patient, 50 years old, presented after 2 years of treatment with quadraparesis secondary to an increase in ossifications of the cervical vertebral ligaments. The neurological damage was resolved after laminectomy, and the patient was then treated for 18 months with doses of 80 mg per week, without recurrence of the bone and muscle signs. The second patient, 26 years old, 78 kg, developed pain and cervical stiffness with pyramidal tract irritation secondary to ossifications of the vertebral ligaments. This improved with a reduction of doses to 80 mg/week, which then, after 6 months of follow‐up, enabled maintained improvement of the bone and muscle pain that was initially obtained. To our knowledge, these are the first reported cases of increased spinal ligamentous ossifications with neurological complications. Biological monitoring in adults does not seem to enable asfotase alfa doses to be adjusted. The levels of serum alkaline phosphatase (ALP) while on the recommended treatment of 1 mg/kg/day are significantly supraphysiological (5000 to 20,000 IU) and the assays of pyrophosphate and pyridoxal phosphate are not correlated with clinical efficacy. In both of our patients, the treatment with 80 mg of asfotase alfa per week, which was proposed after the occurrence of spinal complications, seemed as effective, after a follow‐up of 18 months and 6 months, as the initial treatment for improving the bone and muscle signs, and could be provided as “attack” doses after healing of the pseudoarthroses. © 2021 American Society for Bone and Mineral Research © 2020 The Authors. *JBMR Plus* published by Wiley Periodicals LLC. on behalf of American Society for Bone and Mineral Research.

## Introduction

Hypophosphatasia is a very rare metabolic disease that is caused by a loss of function of the ALPL gene, which codes for the alkaline phosphatase enzyme. Several mutations have been reported, with relationships variable between genotypes and phenotypes.^(^ ^1^, ^2^, ^3^, ^4^ ^)^

The reduction of alkaline phosphatase (ALP) and its bone fraction (bone alkaline phosphatase [BAP]) result in bone mineralization disturbances related to the deficiency of ALP itself and especially to an accumulation of substrates degraded by ALP: pyridoxal 5‐phosphate (PLP), inorganic pyrophosphate (PPi), and phosphoethanolamine.^(^ ^5^, ^6^, ^7^, ^8^, ^9^ ^)^ The clinical signs typically begin in childhood and include, with varying severity, craniostenosis, bone pain, spontaneous fractures with pseudoarthrosis, muscle pain, loss of muscle strength, calcium deposits on the ligaments and on the articular cartilage, and decidual tooth loss. Asfotase alfa is a recombinant human enzyme that has been provided over the past several years as a treatment for hypophosphatasia in children.^(^ ^10^, ^11^, ^12^ ^)^ The indication has been extended to certain forms of adult hypophosphatasia. The efficacy of asfotase alfa was demonstrated in the study by Kishnani and colleagues.^(^ ^13^ ^)^ Other publications concerning isolated cases confirmed this efficacy^(^ ^14^, ^15^ ^)^ and the need to continue treatment given the risk of recurrence of bone signs.^(^ ^16^ ^)^ The same treatment regimen and the same doses were provided in adults and children, although bone remodeling is completely different in adults, and in 2017, Shapiro and Lewiecki pointed out the lack of guidelines in adults.^(^ ^11^, ^17^ ^)^

Apart from skin reactions at the injections site, which are frequent but benign, the side effects concerning three patients from Kishnani and colleagues'^(^ ^13^ ^)^ series were ocular and renal calcifications. The absence of vascular or cardiac calcification is noted in a recent publication.^(^ ^18^ ^)^

We report on the first two case reports of patients treated with 1 mg/kg/day of asfotase alfa, who had increased or modified structuring of spinal ligamentous ossifications with spinal cord compression while on treatment.

## Case reports

Ms. X, born in 1965, 1.60 m, 50 kg, presented with a severe form of hypophosphatasia. Her disease was diagnosed at the age of 20 years in the presence of infantile craniosynostosis, pseudoarthroses, and bone pain.

Testing for the ALPL mutation confirmed the diagnosis (heterozygous: N4611, exon 12).

The pseudoarthroses required rodding of both femurs. Pseudoarthrosis also concerned the right humerus, three ribs, the right fibula, and two metatarsals. Treatment with teriparatide, which was discontinued after 6 months for increased bone pain, was ineffective for consolidating these fractures. The pseudarthroses were associated with chondrocalcinosis of the hips, knees, and shoulders, and with ligamentous ossifications (hyperostosis) in association with intradiscal calcifications.

In September 2016, as part of the AA‐HPP 405 protocol, the patient received treatment with asfotase alfa, 80 mg three times weekly. Before treatment, the total ALP was 7 IU, and the BAP was <2 IU. The serum calcium, serum phosphorous, urinary calcium, and glomerular filtration rate (GFR) were normal, as were the bone turnover markers (CTX: 300 pg/mL [normal = 150–700 pg/mL], osteocalcin: 35 pg/mL [normal = 20–55 pg/mL]). PLP was 150 ng/mL (normal = 15–35 ng/mL).

**Fig 1:**
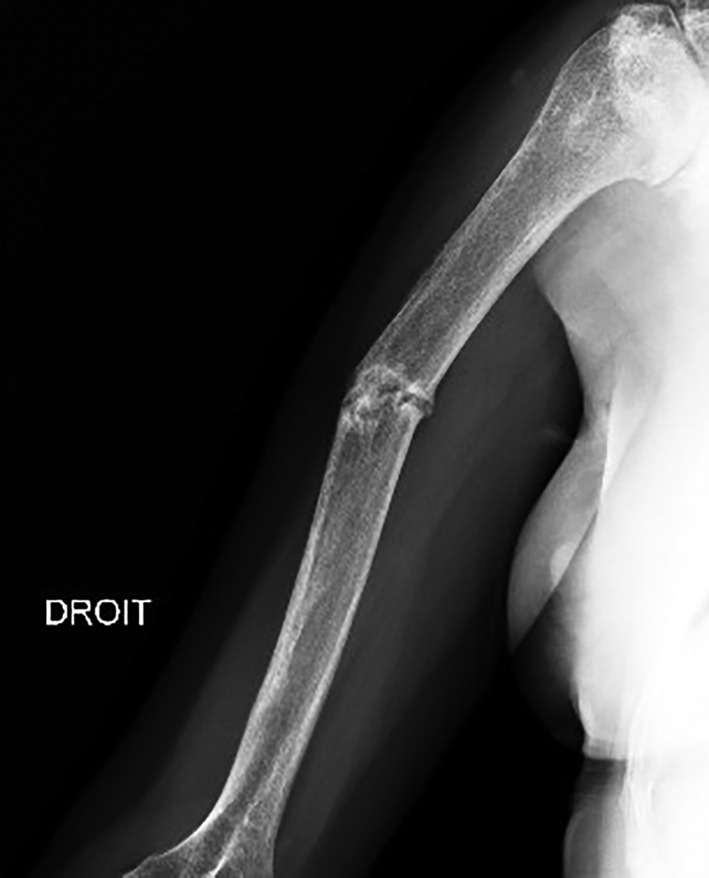
Right side humerus X‐rays: previous pseudarthrosis.

**Fig 2:**
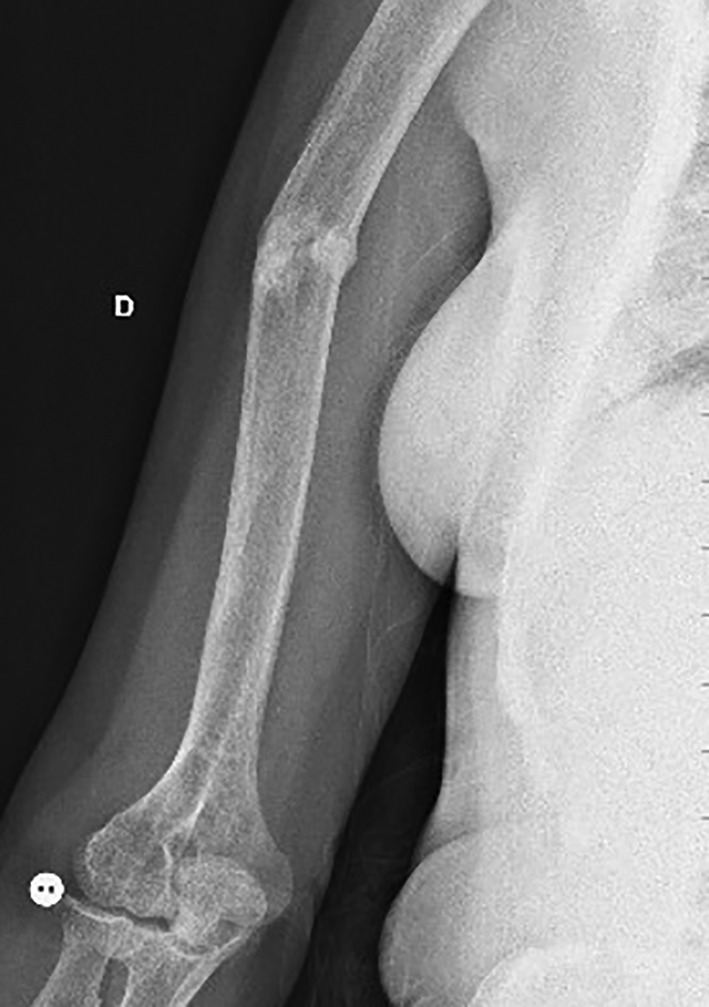
Right side humerus X‐rays after 3 months of asfotase treatment: pseudoarthrosis consolidation.

Bone pain and functional disability improved dramatically in several weeks: the pain visual analogue scale (pain VAS) went from 8/10 to 2/10, and the quality of life visual scale (quality of life VAS) went from 4/10 to 7/10. At 6 months, the pseudoarthroses were consolidated (Figs. 1 and 2).

**Fig 3:**
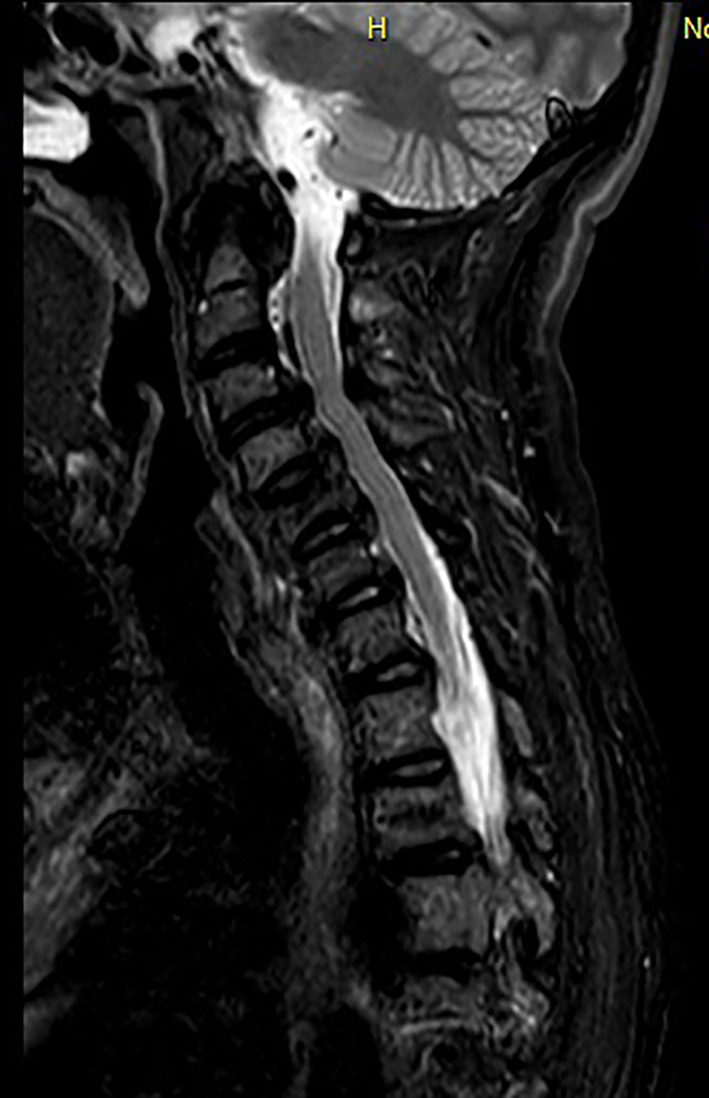
MRI of the cervical spine (March 2017). Sagittal sections, T2‐weighted sequences: extensive ossifications of the posterior longitudinal ligament and the annular ligament with a “crowned dens” appearance.

**Fig 4:**
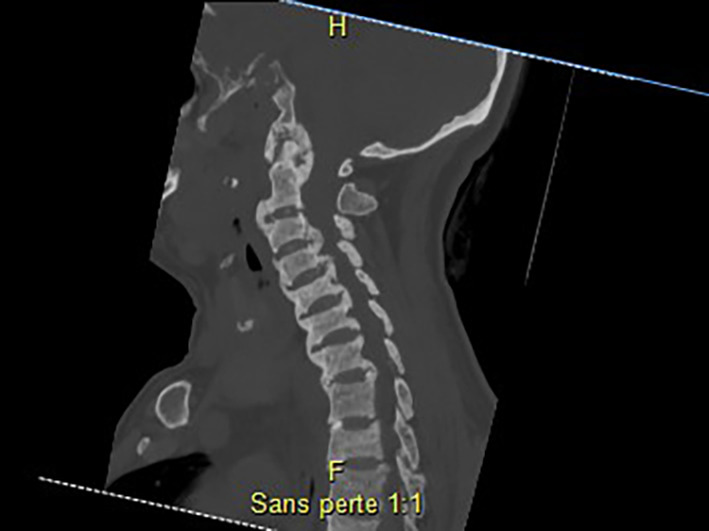
CT scan of the cervical spine (March 2017). Sagittal sections: diffuse bone sclerosis, predominant on the vertebral bodies at the cervical level, sites of biconcave compression. Hyperostosis with enthesopathy and more pronounced discal‐ligamentous ossifications on the anterior and posterior longitudinal ligaments. Ossification of the annular ligament with a “crowned dens” appearance. C_0_–C_1_ and C_1_–C_2_ degenerative modifications.

In March 2017, after 6 months of treatment, the patient presented neck pain with limited movement of the cervical spine (chin‐sternum distance: 8 cm, lateral tilts: 20 degrees, head rotations: 20 degrees), for which a CT scan and an MRI of the cervical spine were done. These exams showed ligamentous ossifications of the anterior longitudinal ligaments (Figs. 3 and 4). C_1_–C_2_ arthrosis was present with crowned dens syndrome. The pain was relieved with diclofenac and physical therapy sessions.

In November 2018, the patient described a sensation of numbness in glove distribution to the upper limb extremities in association with clumsiness with gripping and writing. She also mentioned fatigability of the lower limbs. There was then a pyramidal tract syndrome to all limbs, predominantly in the upper limbs, with abolishment of vibratory and arthrokinetic sensitivity.

**Fig 5:**
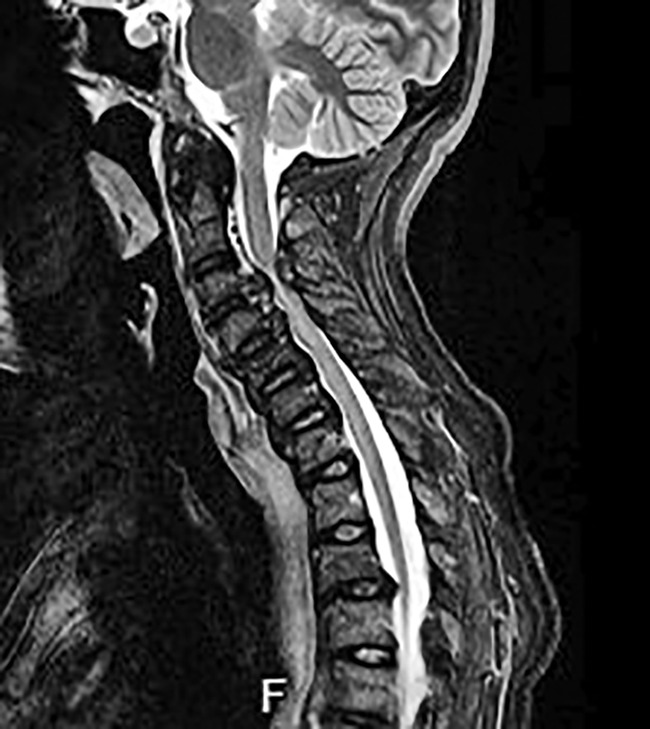
MRI of the cervical spine (December 2018). Sagittal sections, T2‐weighted sequence: Increased ossifications of the posterior longitudinal ligament and the yellow ligaments responsible for narrowing of the vertebral canal, which causes spinal cord compression, as shown by the presence of a C_3_–C_4_ intramedullary hypersignal.

Another cervical MRI showed compression of the cervical spinal cord predominant in C_3_ and C_4_, with structural modification of ossifications of the anterior vertebral ligaments and thickening of the adjacent soft tissue (Fig. 5).

An increase in measurable bone mineral density was noted only in the spine (L_2_–L_4_) due to the rodding of the femurs: 1.245 g/cm^2^ versus 1.038 g/cm^2^ in 2015, which corresponds to a normal *T*‐score at 0.5.

On treatment the ALP was 10.679 IU, BAP was 1400 IU, and the PLP was below the threshold of 5 ng/mL.

Treatment with asfotase alfa was discontinued; a decompressive laminectomy was performed. After stopping the treatment for 21 days, the ALP level was still 4500 IU.

**Fig 6:**
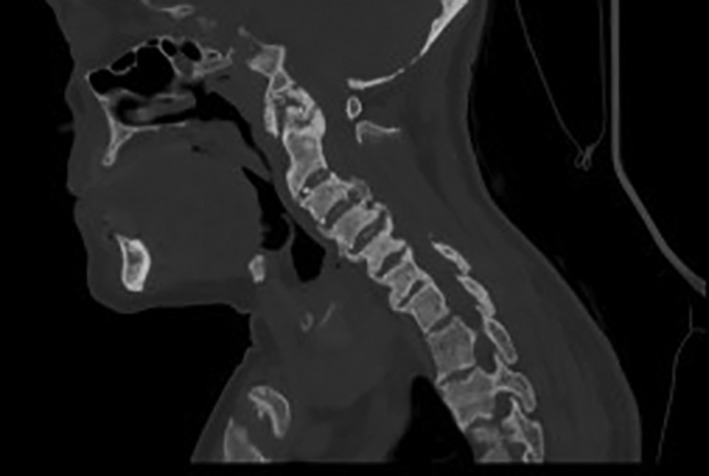
Postoperative CT scan of the cervical spine (March 2019). Sagittal sections: sequelae of posterior laminectomy, decrease in density and thickness of anterior and posterior discal‐ligamentous ossifications.

Two months later, the motor and sensory signs were fully resolved. A CT scan seemed to show modification of the calcific density of the ligamentous ossifications (Fig. 6).

The asfotase treatment was resumed at much lower doses: 80 mg every 21 days. The ALP levels at this dosage were 920 IU 2 days after the injection, 200 IU before that, and PLP was 145 ng/mL the day before the injection.

After 6 months of this treatment regimen, Ms. V said she had recurrence of the initial bone and muscle pain, without fractures, and without radiological modifications.

Asfotase alfa was then administered at 80 mg/week. The patient was seen every month. The pain had disappeared at the end of 2 months on this regimen. The clinical condition was stable after 1 year of follow‐up, with the pain and quality of life VAS identical to those obtained at the start of treatment when the asfotase alfa was 80 mg three times per week. The ALP was 2500 IU 2 days after the injection, 811 IU before that, and the BAP the day before the injection was 250 IU. PLP was 75 ng/mL after the injection, and 155 ng/mL before that.

A CT scan of the chest and an ultrasound exam of the vascular walls did not show calcifications of the coronary arteries or the large vessels. The follow‐up kidney ultrasound was normal, as was the annual ophthalmology follow‐up.

Ms. Y, born in 1990, was managed in the pediatrics department for genetically‐confirmed (heterozygous composite V111M and A115V) hypophosphatasia that had expressed during the neonatal period.

The clinical description of this patient has been described.^(^ ^8^ ^)^ At the end of the pediatric period she developed intense spinal pain, with no origin defined on MRI examinations, and secondarily bilateral subtrochanteric non‐union. Treatment with teriperatide instituted after the end of growth has been shown to be ineffective in pain. She then developed morbid obesity and no longer works. At the age of 27 years, in 2007, we noted an intake of 30 kg in 5 years, a BMI at 39 kg/m^2^, a weight of 78 kg for a height 1.41 m. She continued to suffer from non‐union of the femurs and left wrist, chest deformity, and kyphosis of the back.

Her phosphate‐calcium test was normal, as were the bone turnover markers: CTX 170 pg/mL, osteocalcin: 25 pg/mL. Her ALP was 5 IU, the BAP was below 2 IU, and the PLP was 100 ng/mL.

In September 2016, treatment with asfotase (80 mg, 6/7 days) was started. The patient gradually described decreased bone and muscle pain, and she could walk again with a walker. The pain VAS went from 9/10 to 5/10, and the quality of life VAS from 2/10 to 5/10. The pseudoarthroses consolidated. A simultaneous weight loss of 15 kg was noted.

**Fig 7:**
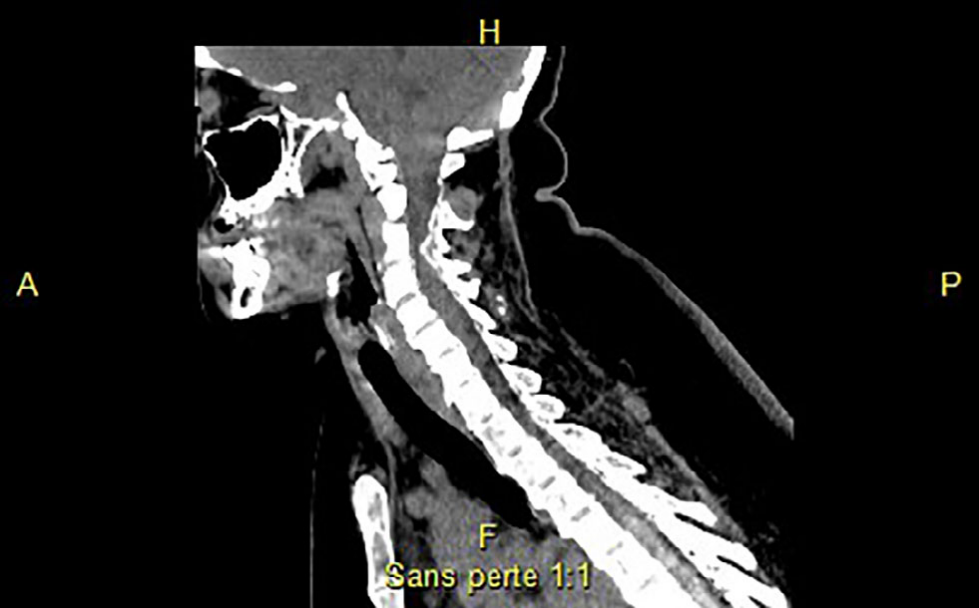
CT scan of the cervical spine, sagittal sections: ossifications of the yellow ligament, more pronounced in C_3_–C_4_.

The treatment was continued at the same doses until January 2019. On treatment the ALP was 20.565 IU, BAP was 6.410 IU, and the pyridoxal phosphate was below 5 ng/mL. The patient reported gradual limitation in mobility of the cervical spine and the lumbar spine. Neck extension was limited (chin‐sternum distance = 10 cm, occiput‐wall distance = 10 cm), as was that of the lumbar spine (Schober's test = 0 cm). Due to considerable pain in these positions the patient could no longer lie down flat on her back and was obliged to sleep in a lateral position despite anti‐inflammatory treatment for 15 days. Although muscle strength was normal, the reflexes to the four limbs were brisk, with spread, with a positive Hoffman's sign bilaterally and Babinski sign on the left. A CT scan done under hypnosis showed major ossification of the posterior longitudinal ligament (Fig. 7). The anesthesiologist refused to sedate the patient to perform an MRI. The follow‐up bone density scan was impossible to perform as the supine position could not be maintained. The follow‐up kidney ultrasound was normal, as was the ophthalmology follow‐up. Treatment with asfotase alfa was then reduced to 80 mg/week. Two months later, Ms. G received daily rehabilitation at a specialized center lasting for 1 month. She was seen again 6 months after the dosage reduction and the rehabilitation. The initial bone pain had not reappeared, there was no pathological fracture (X‐rays unchanged) and the spinal mobility gradually improved to where the supine position could be achieved. The reflexes were still brisk and with spread, but the Hoffman's and Babinski signs were gone. At these doses, on the day following the injection the ALP was 2540 IU, BAP 1200 IU, and PLP 67 ng/mL; on the day before the injection these were, respectively, 1166 IU, 800 IU, and 312 ng/mL.

## Discussion

Our two patients subsequently recovered rapidly from their pseudarthrosis and significantly improved their bone and muscle pain while on asfotase alfa administered at 1 mg/kg/day, but they paradoxically increased and modified the structure of preexisting vertebral and epidural ligamentous ossifications. For one of them this led to cervical spinal cord compression with quadriparesis, which quickly resolved after surgical decompression and temporary discontinuation of the treatment. The other had very painful cervical and lumbar spinal stiffness associated with pyramidal tract irritation, which improved after dose reduction of asfotase alfa to 1 mg/kg per week.

The bone mineralization disorders and extra‐ligamentous and cartilaginous calcium deposits that are responsible for the symptoms of hypophosphatasia are most likely related to the accumulation of PLP and PPi, which the missing ALP cannot degrade.^(^ ^3^, ^4^, ^19^ ^)^ The direct action of ALP seems less likely.^(^ ^9^ ^)^ The phenotypes are quite varied and are associated with genetic mutations that are also quite diverse.^(^ ^1^, ^2^, ^3^ ^)^ The pathophysiology of the muscle signs is poorly understood; however, the PLP could participate in their pathophysiology.^(^ ^7^ ^)^

Antiresorptive agents, biphosphonates or denosumab, worsen bone lesions and are contraindicated^(^ ^20^ ^)^ in osteoporosis secondary to hypophosphatasia. Some patients could be successfully treated with teriparatide, though this may be ineffective in other cases, as it was for our two patients.^(^ ^21^ ^)^

The efficacy of asfotase alfa (1 mg/kg/day 6 times weekly or 2 mg/kg/day 3 times weekly) has been clearly demonstrated in infantile hypophosphatasia. Compared with the natural history of the disease, it improves life expectancy: 95% survival at 1 year versus 42% without treatment, 84% at 5 years versus 27% without treatment.^(^ ^11^, ^12^ ^)^ Asfotase alfa improved growth, respiratory function, and muscular function. The same doses were provided in adults, and one study by Kishnani and colleagues.^(^ ^13^ ^)^ demonstrated more moderate efficacy in adults. Thirteen patients, with a mean age of 55 years, were treated for 5 years and compared with six patients who had been treated with placebo. There was no significant difference in the densitometry measurements or in the histomorphometry parameters in the different groups. The walking perimeter increased significantly but moderately, going from 350 to 450 m in the group treated with asfotase alfa. Muscle strength improved but pain did not. The decrease in the PPi level was not significant: +0.2 μm (−6.8 to +1.1) in the control group versus –2.2 μm (−4.4 to +0.3) in the treated group (*p* = 0.071), whereas that of the PLP was significant in patients treated with asfotase: +11 ng/mL (−374 to +346) versus −245 ng/mL (−1467 to −17.2) (*p* = 0.028). The ALP levels were very high: 6819 IU (3047–12,630 IU) in patients treated.

The side effects were mainly benign or moderate skin reactions (erythema, pruritis, skin discoloration, lipodystrophy at the injection sites). Two patients had ocular calcifications and one had nephrocalcinosis.

Based on this study therefore, treatment with asfotase alfa was authorized in France in adult patients whose symptoms of hypophosphatasia had started in their childhood. The proposed doses were those used in this study. Our first patient, weighing 50 kg, was treated with 80 mg of asfotase alfa subcutaneously three times weekly. The second patient, 80 kg, was treated with 80 mg subcutaneously 6 out of 7 days. These patients had a rapid decrease in their bone pain, within 6 months, and there was consolidation of the pseudoarthroses, which concerned in one patient the right fibula, the right humerus, the ribs, the metatarsals, and for the other, both femurs and the left wrist. The neurological disorders started in the first patient after 2 years of treatment. Morphological exams showed a significant increase in epidural and vertebral ligamentous ossifications which caused spinal compression. The treatment was then discontinued for 6 weeks, the time of the surgery and the time for neurological recovery; it was then resumed at lower doses: 80 mg every 21 days for 6 months, and then 80 mg every week. During the treatment with 80 mg every 21 days, the bone pain recurred; it then disappeared and did not return at the dose of 80 mg/week after 1 year of follow‐up. In the second patient, very painful cervical and lumbar stiffness occurred gradually after a year and a half of treatment, which prevented the supine position. Cervical spine and lumbar spine mobility was nearly nonexistent. The treatment was decreased to 80 mg/week, with gradual improvement of the pain and mobility in 6 months. It is possible that the reduction in the dosage of afostase led to a change in the structure of the ligamentous ossifications, explaining the improvement in clinical signs without surgery. For this patient, however, the lack of prior CT scan or MRI did not allow this hypothesis to be formally confirmed.

A panel of experts that met to define the monitoring of patients with treated hypophosphatasia pointed out the difficulty of current PPi determination and the interferences between recombinant ALP in the tube and the PLP that degrades it.^(^ ^22^ ^)^ There is therefore no recommended biological follow‐up, except for ALP determinations, only to verify compliance with treatment. Experts recommend clinical follow‐up based on the assessment of bone pain, muscle strength, functional parameters, and screening for ophthalmological and renal complications with repeated annual ultrasound examinations.^(^ ^23^ ^)^

Our two case reports demonstrate potential toxicity of the treatment at the doses recommended in the study by Whyte and Kishnani, paradoxically resulting in hyperostosis with enthesopathy and more marked discal‐ligamentous ossification on the anterior and posterior longitudinal ligaments.^(^ ^13^ ^)^ The mechanism for this worsening of ectopic ossifications is unknown, which is also the case, in some treated patients, for ocular or renal ossifications.

Our two case reports could lead to the suggestion to perform an MRI of the spine before starting treatment with aafostase, then every year if ligament ossifications are found on the first MRI.

In addition, after a follow‐up of 18 months for one and 9 months for the other, our two case reports show the clinical efficacy of much lower doses of asfotase: 1.5 mg/kg per week for the first; 1 mg/kg per week for the second. At these doses, the ALP level was 2000 and 2500 IU/L the day after the injection, and 1000 and 1200 IU/L until the day before the injection. The PLP varied from 79 ng/mL after the injection to 155 ng/mL before the injection for the first patient, and from 67 to 312 ng/mL for the second patient.

It seems conceivable to us to provide a 6‐month “attack” treatment at doses of 1 mg/kg/day, as recommended by Kishnani and Whyte, to heal the pseudarthrosis: radiological target very easy to define, followed by a maintenance treatment at much more moderate doses, of around 1 to 1.5 mg/kg per week, to be adjusted based on whether or not there are bone and/or muscular clinical signs, maintaining ALP levels between 1000 and 2500 IU/L.^(^ ^13^ ^)^ The immunogenicity of such a dose spacing strategy should be assessed.

This strategy would allow the cost of treatment, borne in France by the French health insurance fund, to be considerably reduced, which for our two patients was initially 1,000,000 euros per year for the first and 1,500,000 euros for the second.

Considering the rarity of this condition, observational multicenter studies should be conducted to confirm this proposal.

In addition, careful screening of the ALP levels in assessments of idiopathic osteoporosis has enabled in the past several years the diagnosis of subclinical forms of hypophosphatasia, genetically confirmed, in which osteoporosis, expressed through “classic” mainly vertebral fractures, is the only complication.^(^ ^2^ ^)^ In these benign forms, probably 10 to 20 times more common than the severe forms of pediatric onset, bisphosphonates and denosumab are theoretically contraindicated, and teriparatide or romososumab, although they are effective, can only be administered over a limited period (12 to 24 months). It seems inconceivable, due to the public health cost that this would entail, to consider treatments with asfotase alfa at doses of 1 mg/kg/day. Prospective, randomized, multicenter studies are essential in this indication for defining the minimum effective, and nontoxic dose of asfotase alfa.

## Conclusion

Treatment of the serious forms of hypophosphatasia in adults at the recommended doses of 1 mg/kg/day, based on the studies by Whyte, can lead to modifications in the volume and structure of spinal ossifications with spinal cord compression.^(^ ^13^ ^)^ Following consolidation of pseudarthroses, long‐term treatment with asfotase alfa may be considerably reduced: doses of 1 and 1.5 mg/kg per week were sufficient in our two patients for maintaining a satisfactory bone and muscle status.

## Disclosures

The authors declare that they have no conflict of interest about this work.

## AUTHOR CONTRIBUTIONS

**Michel Laroche:** Conceptualization; writing‐original draft; writing‐review and editing. **Guillaume Couture:** Visualization. **Marie Faruch:** Supervision. **Adeline Ruyssen‐Witrand:** Supervision. **Valérie Porquet‐Bordes:** Supervision. **Jean‐Pierre Salles:** Supervision. **Yannick Degboe:** Conceptualization; supervision; validation.

### PEER REVIEW

The peer review history for this article is available at https://publons.com/publon/10.1002/jbm4.10449.
